# Utility of Cry1Ja for Transgenic Insect Control

**DOI:** 10.3390/toxins16090384

**Published:** 2024-09-04

**Authors:** John P. Mathis, Catherine Clark, Amit Sethi, Benchie Ortegon, Gilda Rauscher, Russ Booth, Samuel Coder, Mark E. Nelson

**Affiliations:** Corteva Agriscience, Indianapolis, IN 46268, USA; catherine.clark@corteva.com (C.C.); amit.sethi@corteva.com (A.S.); benchie.ortegon@corteva.com (B.O.); gilda.rauscher@corteva.com (G.R.); russ.booth@corteva.com (R.B.); sam.coder@corteva.com (S.C.)

**Keywords:** *Bacillus thuringiensis*, Cry1J protein, competitive binding assay, insecticidal protein bioassay, in planta assay, *Helicoverpa zea*, *Spodoptera frugiperda*, *Chrysodeixis includens*

## Abstract

Insect control traits are a key component of improving the efficacy of insect pest management and maximizing crop yields for growers. Insect traits based on proteins expressed by the bacteria *Bacillus thuringiensis* (Bt) have proven to be very effective tools in achieving this goal. Unfortunately, the adaptability of insects has led to resistance to certain proteins in current commercial products. Therefore, new insecticidal traits representing a different mode of action (MoA) than those currently in use are needed. Cry1Ja has good insecticidal activity against various lepidopteran species, and it provides robust protection against insect feeding with in planta expression. For Bt proteins, different MoAs are determined by their binding sites in the insect midgut. In this study, competitive binding assays are performed using brush border membrane vesicles (BBMVs) from *Helicoverpa zea*, *Spodoptera frugiperda*, and *Chrysodeixis includens* to evaluate the MoA of Cry1Ja relative to representatives of the various Bt proteins that are expressed in current commercial products for lepidopteran insect protection. This study highlights differences in the shared Cry protein binding sites in three insect species, Cry1Ja bioactivity against Cry1Fa resistant FAW, and in planta efficacy against target pests. These data illustrate the potential of Cry1Ja for new insect trait development.

## 1. Introduction

The deployment of Bt-derived insect control traits has been used successfully to reduce insect damage in crops for years. In 2024, Bt corn accounted for 86% of corn acreage grown in the USA and Bt cotton accounted for 90% of cotton acreage grown in the USA [1,2]. This high rate of utilization has been attributed to the positive economic and environmental benefits of Bt technology outweighing the negative factors. The financial benefits include such factors as higher crop yields, reduced risk of insect-induced fungal contamination, and lower input costs related to reduced labor and insecticide applications [3,4]. The beneficial economic factors outweigh the negative factors associated with Bt crops, which include the increased seed cost, possible limits on export market requirements, and negative public perception [4,5]. The environmental risks that were once proposed to be associated with Bt crops, such as negative impacts on beneficial insect populations, have been shown to be false [5]. In contrast, reduced insecticide use also results in lower CO_2_ emissions from lower fuel consumption and an increase in beneficial insect populations [3]. Higher crop yields also help to conserve biodiversity by reducing the amount of land needed to produce the same amount of grain [6]. The food derived from GM crops has been shown to pose no sustainable risk to humans or livestock [4,7].

The benefits of an insect trait can be limited by the development of resistance [3]. Insect resistance management (IRM) practices are used to delay or limit the development of resistant insect populations. Bt trait deployment has consisted of traits that rely on the expression of a single protein and traits that result in the co-expression of more than one protein (gene pyramiding). The first generation of Bt traits were single active traits that followed the high dose/refuge strategy. This strategy relies on the assumptions that resistance is recessive, the resistance allele frequency is low in the general population, and the trait is high dose, meaning that >99.9% of susceptible individuals do not survive exposure to the Bt crop. The high dose strategy has been effective at promoting the durability of some traits [3,8]. In the United States, the use of insect refuges has been required by the US Environmental Protection Agency for the commercialization of Bt crops. Refuge promotes the production of susceptible insects to serve as a mate for insects that survive exposure to a Bt crop that could harbor resistance alleles. The high dose/refuge strategy would be most effective for proteins that meet the high dose criteria.

The gene-pyramiding strategy involves the use of at least two genes encoding insecticidal molecules that work through different mechanisms against the same target insect. In the case of genes that encode insecticidal proteins, this translates to genes that encode proteins having different sites of action. The main advantage of the gene-pyramiding strategy is “redundant killing”, where an insect resistant to one protein is killed by the other protein encoded by the gene pyramid. Thus, insects possessing homozygous resistant alleles for both proteins would be exceedingly rare in the population. Mathematical modeling of a gene pyramided trait compared to a single gene trait indicates a significant delay in the onset of resistance in the population [9,10]. Another advantage of gene pyramiding is that non-high-dose traits can benefit from pyramiding with similar proteins [3]. New proteins that do not meet the high dose criteria could be part of a gene-pyramiding design strategy to bolster resistance management options.

Despite efforts to delay the development of resistance, the number of field-evolved insect resistance populations has continued to increase in parallel with the increased planting of Bt crops around the world [3,11,12]. As of 2016, the number of reported field-evolved insect resistance populations has increased from 3 in 2008 to 16 in 2016 [11]. The mechanisms of Bt resistance in these field-evolved insect resistance populations often involve changes to protein binding to their target receptors within the larval gut [13]. The continuous evolution of resistance necessitates a constant effort to develop new insecticidal traits with novel site of actions relative to those utilized by currently deployed traits.

Therefore, we have investigated Cry1Ja’s effectiveness in controlling certain lepidopteran species as a new insecticidal trait. Specifically, this study evaluated the ability of Cry1Ja to interact at various Cry protein binding sites in three insect species, *Chrysodeixis includens* (soybean looper; SBL), *Helicoverpa zea* (corn earworm; CEW), and *Spodoptera frugiperda* (fall armyworm; FAW), its bioactivity against Cry1Fa resistant FAW, and its in-planta efficacy. This study took advantage of two mutated forms of Cry1J variant Cry1JDP166 proteins, Cry1JP578V and Cry1JPS1 [14] (see Section 5.1 and Section 5.4, respectively). Cry1JP578V is a Cry1J protein that showed increased stability in vitro when subjected to trypsin processing to simulate proteolytic activation in vivo to prepare it for binding evaluations. A structural model of Cry1JP578V can be found in Figure S1 [15]. Cry1JPS1 is a Cry1J variant of Cry1JDP166 that has previously been shown to have activity against SBL, CEW and FAW, and it was used in this study to demonstrate in planta efficacy [14].

## 2. Results

### 2.1. Characterization of Cry1JP578V Binding in Three Lepidopteran Species

**Figure 1:**
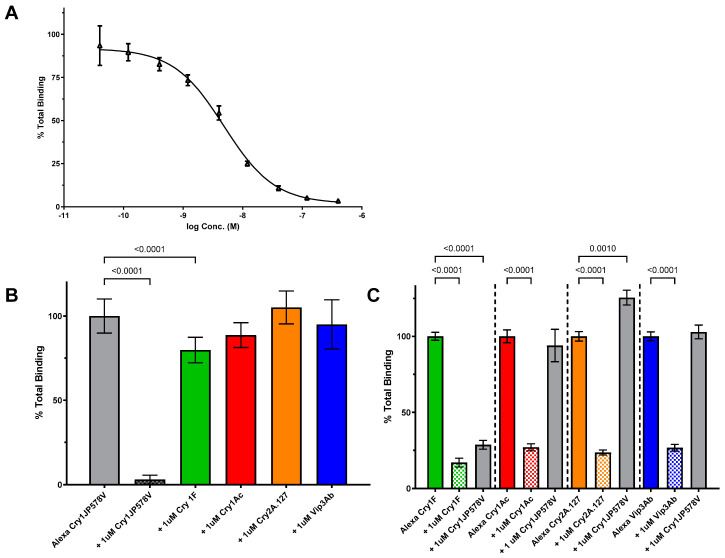
Cry1JP578V competition binding assays in SBL. (**A**) Binding of Alexa-labeled Cry1JP578V to SBL BBMV illustrated by homologous competition. BBMVs (30 µg) were incubated with Alexa-Cry1JP578V (2.5 nM) in the absence (“Total”) and presence of unlabeled Cry1JP578V protein. The graphs plot the average densitometry values measured from images taken of in-gel fluorescence (see Section 5.3) for each binding reaction and normalized to the values measured in the absence of competitor, i.e., “% Total Binding”. (**B**) Heterologous competition between Alexa-labeled Cry1JP578V and representatives of trait proteins with SBL BBMV show “% Total Binding” of Alexa-Cry1JP578V (2.5 nM) to SBL BBMV (30 μg) in the absence (n = 24) and presence of 1 μM unlabeled Cry1JP578V (n = 24), Cry1Ac, Cry1Fa, Cry2A.127, and Vip3Ab (n = 6). (**C**) Reciprocal competition (n = 6) for 50 nM Alexa Cry1Ac and 5 μg SBL BBMVs; 5 nM Alexa Cry1Fa and 60 μg SBL BBMVs; 20 nM Alexa Cry2A.127 and 30 μg SBL BBMVs; 10 nM Alexa Vip3Ab and 2 μg SBL BBMVs. All the error bars reflect the standard deviation. *p*-values < 0.01 are indicated by a bracket above the compared columns. Statistical analysis was performed with one-way ANOVA with Tukey’s post hoc test for Panel (**B**) and with one-way ANOVA with Sidak’s post hoc test for Panel (**C**).

Characterization of Cry1JP578V binding in SBL, CEW and FAW BBMVs was accomplished with a combination of homologous and heterologous competition binding assays. In SBL, the homologous competition assays showed a displacement curve with an EC_50_ value of 4.8 nM (n = 4, 95% CI [3.2, 7.2]) (Figure 1A). The heterologous competition assays indicated that a saturating concentration (1 µM; determined from homologous competition) of unlabeled Cry1Ac, Cry2A.127 (a variant of Cry2Ab), or Vip3Ab was unable to significantly compete with Alexa Cry1JP578V binding to SBL BBMV. Cry1Fa was able to compete with Alexa Cry1JP578V binding to SBL BBMV to a small (20% of total) but significant (*p* < 0.001) amount (Figure 1B). The reciprocal binding assays showed that saturating concentrations (1 µM) of unlabeled Cry1JP578V did compete with the binding of Alexa-labeled Cry1Fa and did not compete with the binding of Alexa-labeled Cry1Ac, Cry2A.127 or Vip3Ab (Figure 1C). These results indicate that Cry1JP578V completely shares binding sites with Cry1F, and Cry1F partially shares binding sites with Cry1J. In addition, these results show that Cry1JP578V does not share binding sites with Cry1Ac, Cry2A.127 or Vip3Ab in SBL. In a further analysis of the Cry1J site of action in CEW and FAW, Cry1A.88 (a variant of Cry1Ab) was used to examine the Cry1J interaction at Cry1A’s sites of action. Cry1Ac was used to examine the interaction between Cry1J and Cry1A’s sites of action in SBL due to our inability to detect Cry1A.88 binding in SBL.

**Figure 2:**
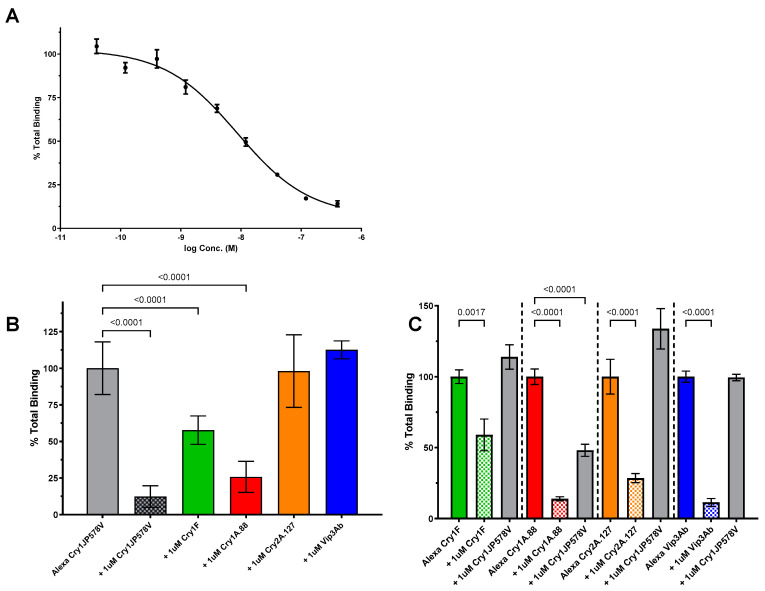
Cry1JP578V competition binding assays in CEW. (**A**) Binding of Alexa-labeled Cry1JP578V to CEW BBMV illustrated by homologous competition. BBMVs (30 µg) were incubated with Alexa-Cry1JP578V (5 nM) in the absence (“Total”) and presence of unlabeled Cry1JP578V protein. The graphs plot the average densitometry values measured from images taken of in-gel fluorescence (see Section 5.3) for each binding reaction and normalized to the values measured in the absence of the competitor, i.e., “% Total Binding”. (**B**) Heterologous competition between Alexa-labeled Cry1JP578V and representatives of trait proteins with CEW BBMV show “% Total Binding” of Alexa-Cry1JP578V (5 nM) to CEW BBMV (30 μg) in the absence and presence of 1 μM unlabeled Cry1JP578V, Cry1Ac, Cry1Fa, Cry2A.127, and Vip3Ab (n = 6–15). (**C**) Reciprocal competition (n-6) for 2.5 nM Alexa Cry1A.88 and 25 μg CEW BBMVs; 100 nM Alexa-Cry1Fa and 1 μg CEW BBMVs; 10 nM Alexa-Cry2A.127 and 60 μg CEW BBMVs; 10 nM Alexa-Vip3Ab and 40 μg CEW BBMVs. All the error bars reflect the standard deviation. *p*-values < 0.01 are indicated by a bracket above the compared columns. Statistical analysis was performed with one-way ANOVA with Tukey’s post hoc test for Panel (**B**) and with one-way ANOVA with Sidak’s post hoc test for Panel (**C**).

The homologous competition of Alexa Cry1JP578V in CEW exhibited a displacement curve with an EC_50_ value of 8.6nM (n = 4, 95% CI [5.4, 13.6]) (Figure 2A). The heterologous competition assays indicated that a saturating concentration (1 µM) of unlabeled Cry2A.127 or Vip3Ab was unable to compete with the Alexa Cry1JP578V binding to CEW BBMV (Figure 2B). The reciprocal binding assays showed a saturating concentration (1 µM) of unlabeled Cry1JP578V did not compete with the Alexa-labeled Cry2A.127 or Alexa-labeled Vip3Ab (Figure 2C). Cry1Fa was able to partially compete with the Alexa Cry1JP578V binding to CEW BBMV to an incomplete level but a significant (*p* < 0.001) amount (Figure 1B). However, the reciprocal binding assays showed that saturating concentrations (1 µM) of unlabeled Cry1JP578V did not compete with the binding of Alexa-labeled Cry1Fa (Figure 2C). Cry1A.88 could displace Alexa-Cry1JP578V completely and the reciprocal Alexa-Cry1A.88 binding assay showed Cry1JP578V partially competed with the Cry1A.88 binding (Figure 2C). These results indicate that Cry1JP578V partially shares the Cry1A.88 binding site and Cry1A.88 completely shares the Cry1J binding site. In contrast, Cry1F partially shares the Cry1J binding site, but Cry1JP578V does not share the Cry1F binding site. Finally, these results show that Cry1JP578V does not shared binding sites with Cry2A.127 or Vip3Ab in CEW.

**Figure 3:**
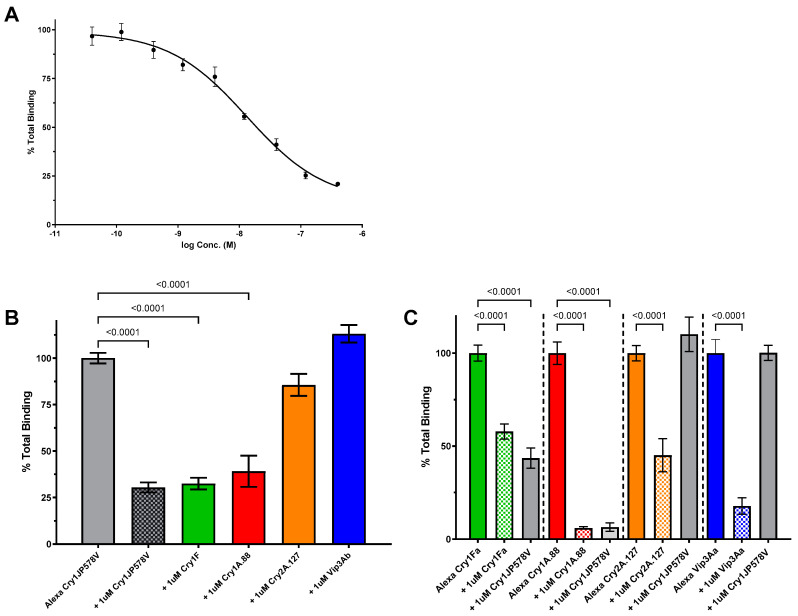
Cry1JP578V competition binding assays in FAW. (**A**) Binding of Alexa-labeled Cry1JP578V to FAW BBMV illustrated by homologous competition. BBMVs (10 µg) were incubated with Alexa-Cry1JP578V (5 nM) in the absence (“Total”) and presence of unlabeled Cry1JP578V protein. The graphs plot the average densitometry values measured from images taken of in-gel fluorescence (see Section 5.3) for each binding reaction and normalized to the values measured in the absence of the competitor, i.e., “% Total Binding”. (**B**) Heterologous competition between Alexa-labeled Cry1JP578V and representatives of trait proteins with FAW BBMV show “% Total Binding” of Alexa-Cry1JP578V (5 nM) to FAW BBMV (10 μg) in the absence and presence of 1 μM unlabeled Cry1JP578V, Cry1Ac, Cry1Fa, Cry2A.127, and Vip3Ab (n = 3–12). (**C**) Reciprocal competitions (n = 3–6) for 2.5 nM Alexa-Cry1A.88 and 25 μg FAW BBMVs; 10 nM Alexa-Cry1Fa and 40 μg FAW BBMVs; 10 nM Alexa-Cry2A.127 and 40 μg FAW BBMVs; 10 nM Alexa Vip3Ab and 2 μg FAW BBMVs. All the error bars reflect the standard deviation. *p*-values < 0.01 are indicated by a bracket above compared columns. Statistical analysis was performed with one-way ANOVA with Tukey’s post hoc test for Panel (**B**) and with one-way ANOVA with Sidak’s post hoc test for Panel (**C**).

In FAW, the Alexa-Cry1JP578V homologous competition revealed a displacement curve with an EC_50_ value of 13 nM (n = 4, 95% CI [6.9, 25.5]) (Figure 3A). The heterologous competition assays indicated that a saturating concentration (1 µM) of unlabeled Cry2A.127 or Vip3Ab was unable to compete with the Alexa-Cry1JP578V binding in FAW BBMV (Figure 3B). The reciprocal binding assays showed saturating concentrations (1 µM) of unlabeled Cry1JP578V did not compete with the Alexa-labeled Cry2A.127 or Alexa-labeled Vip3Ab. However, Cry1A.88 and Cry1Fa could displace Alexa-Cry1JP578V, and the reciprocal competitions of Alexa-Cry1A.88 and Alexa-Cry1Fa binding showed that Cry1JP578V competed with both the Cry1A.88 and Cry1Fa binding completely (Figure 3C). These data demonstrate the sharing of the FAW Cry1JP578V binding sites with Cry1A.88 and Cry1Fa, but they are not shared with Cry2A.127 or Vip3Ab.

### 2.2. Activity of Cry1J against Cry1Fa-Resistant Spodoptera frugiperda

**Figure 4:**
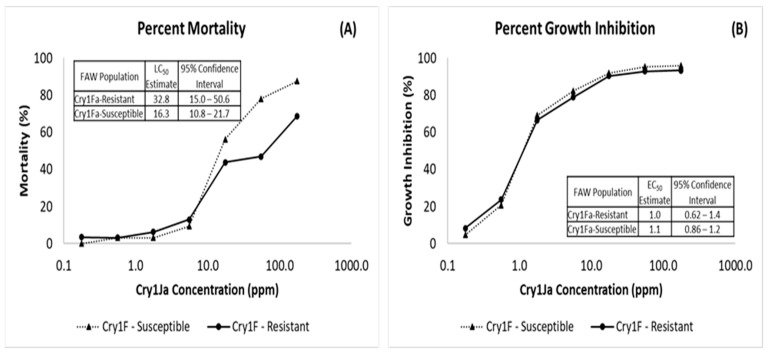
Evaluation of cross-resistance between Cry1JP578V and Cry1Fa using Cry1Fa-resistant and susceptible FAW populations. (**A**) Concentration–mortality response and LC_50_ of Cry1Fa-resistant and susceptible FAW populations to Cry1JP578V. (**B**) Concentration–growth inhibition response and EC_50_ of Cry1Fa-resistant and susceptible FAW populations to Cry1JP578V. Mortality and growth inhibition were assessed after 4 days feeding in artificial diet bioassays.

To evaluate the relevance of the shared Cry1JP578V binding site to toxicity in FAW and to demonstrate the effectiveness of Cry1JP578V in relation to Cry1Fa-resistant FAW [16], artificial diet-based bioassays with the Cry1JPS1 protein were conducted on susceptible and Cry1Fa-resistant FAW. No significant difference was found between the Cry1JPS1 LC_50_ and EC_50_ values for Cry1Fa-resistant and Cry1Fa-susceptible populations (Figure 4A,B). These data indicate that no cross-resistance exists between Cry1JPS1 and Cry1Fa in resistant FAW and suggest that the unshared Cry1JP578V binding site contributes substantially to toxicity.

### 2.3. In Planta Efficacy of Cry1J against Three Lepidopteran Species

**Figure 5:**
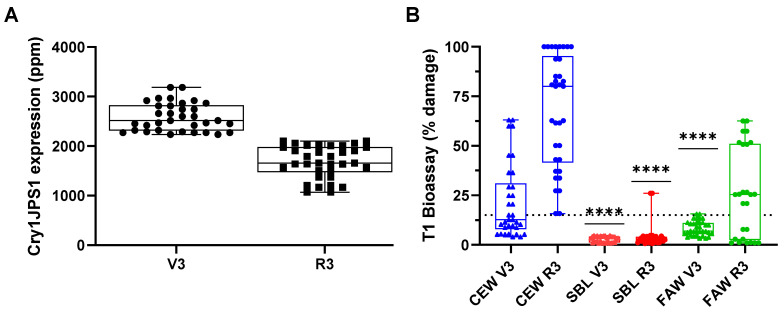
In planta efficacy evaluation of Cry1JPS1. (**A**) Cry1JPS1 protein accumulation in T1 homozygous soybean plants. Samples analyzed were V3- and R3-stage leaf tissue. Protein accumulation was determined by mass spectrometry as described before [17]. The box represents the 25th to 75th percentiles, the line reference marker illustrates the median values, the whiskers show the min to max of the data range for each sample type, and each individual value is a point superimposed on the graph. (**B**) Cry1JPS1 in planta efficacy against three lepidopteran pests. The data show the percent damage to the soybean plants tested [18]. Leaf disk samples at the V3 and R3 stages were infested with neonates for each one of the three insects (CEW, SBL and FAW). The box represents the 25th to 75th percentiles, the line reference marker illustrates the median values, the whiskers show the min to max of data range for each sample type, and each individual value is a point superimposed on the graph. The dash line in Panel (**B**) shows 15% tissue damage. *p*-values < 0.0001 are indicated by asterisks above the columns for values below 15%. Statistical analysis was performed with a one-sample *t* test for Panel (**B**).

The Cry1JPS1 efficacy was evaluated with leaf tissue taken from the R3 and V3 growth stages of T1 homozygous plants. Cry1JPS1 accumulated at similar levels in both the V3 and R3 growth stages, 2818 and 1822 ppm, respectively (Figure 5A). The in planta Cry1JPS1 expression was able to control the feeding of SBL as evaluated as the median tissue damage value at both growth stages being under 15%. FAW feeding may have been controlled at V3 but not at the R3 life stage as compared using the median tissue damage values of <15% and >15%, respectively. In contrast, CEW feeding on Cry1JPS1-expressing plant tissue was far less controlled as compared to the other insect species evaluated (Figure 5B). These results show that Cry1JPS1 may have selective control of only two of the three lepidopteran species evaluated at certain plant life stages at the protein accumulation levels that were evaluated.

## 3. Discussion

Bt proteins have been widely and successfully used as insecticidal traits in cotton, potato, and corn for approximately two decades and in soybean more recently [1,2]. However, the constant evolution of insect resistance to Bt traits necessitates a continuing effort to identify and deploy new proteins with differing sites of action than the proteins currently deployed. Cry1Ja was first shown to have activity against *Plutella xylostella* and *Pectinophora gossypiella* [19,20,21]. These early and subsequent studies [22,23] reported Cry1Ja activity against a wide range of lepidopteran species in artificial diet bioassays, but none reported in planta efficacy. We sought to demonstrate that Cry1J could be efficacious in controlling feeding by key lepidopteran crop pests. Our results show that Cry1J could be expressed in soybean and accumulated at levels sufficient to control the feeding of FAW and SBL, but not CEW. As far as we are aware, this is the first published report of the in planta efficacy for Cry1J against lepidopteran crop pests.

A key objective of the current study was to determine if Cry1Ja shares binding sites with other Cry proteins that are produced by insect control traits in corn and soybean. To achieve this objective, we developed a Cry1Ja binding assay for key crop pests, CEW, FAW and SBL, and based on those results, we developed a binding site model for each. The Cry1Ja binding affinities were similar for each insect; 4.8, 8.6 and 13 nM for SBL (Figure 1), CEW (Figure 2) and FAW (Figure 3), respectively. Biotin-labelled labeled Cry1J has been used to demonstrate that Cry1Ja, Cry1Ab and Cry1Ac shared binding sites across several lepidopteran species, but no measure of affinity was reported [23].

Although the Cry1Ja binding affinities in the three insects were similar, the pattern of shared binding sites with the current trait proteins differed for each. For SBL, Cry1Ja did not share any binding sites with the other proteins examined (Figure 1). Previous reports on Cry protein binding in SBL were mainly focused on Cry1Ac binding and its extent of shared sites with other Cry proteins, such as Cry1Fa, Cry1Ca, Cry1E and Cry2A [24,25,26]. These reports stated that Cry1Ca, Cry1E and Cry2A do not share binding sites with Cry1Ac or Cry1Fa. However, Bel et al. [24] showed that Cry1Ac and Cry1Fa partially share binding sites. Our current results agree with these previous reports in that Cry2A does not share binding with Cry1Ja similar to other Cry1 toxins. Cry1Fa does partially share binding sites with Cry1J, but Cry1Ja completely shares binding sites with Cry1Ac. Our data for Cry1J add to the site of action characterization for Cry proteins that are active against SBL.

**Figure 6:**
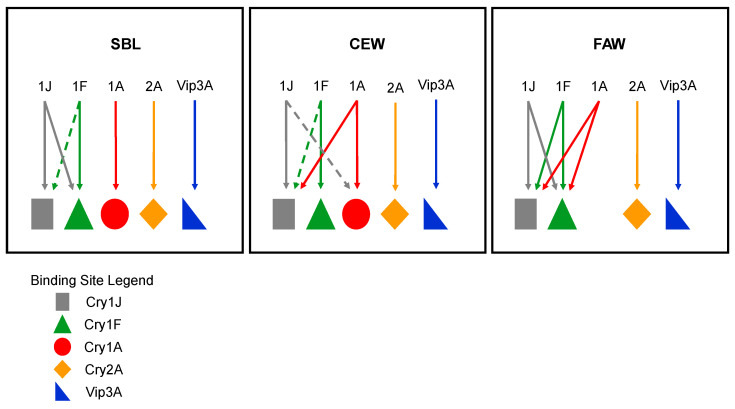
Models depicting the binding site relationships between Cry1JP578V and the various proteins evaluated in each insect. Solid lines indicate complete binding site sharing and dashed lines indicate partial binding site sharing. Binding sites are represented by different geometric shapes and proteins are indicated the letters and numbers. In SBL BBMVs, Cry1JP578V only shares binding sites Cry1Fa but not with Cry2A.127 and Vip3A. In CEW BBMVs, Cry1JP578V, Cry1Fa and Cry1A.88 shared binding sites, while Cry2A.127 and Vip3A have completely independent binding sites. In FAW BBMVs, Cry1JP578V shares binding sites with Cry1A.88 and Cry1Fa, but also has an independent binding site that was not shared with Cry2A.127 and Vip3A.

For CEW, the Cry1Ja binding sites were completely shared with Cry1A.88 (a Cry1Ab variant), but the Cry1A.88 binding sites were only partially shared with Cry1Ja, indicating that Cry1Ja binds to a subset of Cry1Ab binding sites. The Cry1Ja binding sites were partially shared with Cry1Fa, but the Cry1Fa binding sites were not shared with Cry1Ja indicating that Cry1Fa binds to a subset of Cry1Ja binding sites. Furthermore, our data showed that Cry1Ja does not share binding sites with Cry2A.127 (Cry2A variant) or Vip3A (Figure 6). A previous study found that Cry1J shared binding sites with both Cry1Ac and Cry1Fa in CEW [27]. The sharing of binding sites with Cry1Ac is in agreement with our data when taken in the context of Cry1Ac and Cry1Ab having been reported to share binding sites in CEW [28] and our own testing showed that Cry1Ac and Cry1A.88 share binding sites (unpublished data). However, the relationship between Cry1Ja and Cry1Fa in our results differs from the previous study [27]. This discrepancy could be due to a differences in binding assay conditions (e.g., BBMV preparations, buffers, labelling method, etc.) or variations in receptor properties due to differences in the insect strains and rearing methods used [29].

Cry1Ja binding in FAW was less complex than in the other insect species evaluated. Our results showed that the Cry1Ja binding sites were completely shared with Cry1A.88 and Cry1Fa, and the Cry1A.88 and Cry1Fa binding site were completely shared with Cry1Ja (Figure 6). This indicates that Cry1Ja binds to all the Cry1Ab or Cry1Fa receptors and conversely Cry1Ab or Cry1Fa binds to all the Cry1Ja receptors. The shared receptors between Cry1Ja and Cry1Ab or Cry1Fa agree with previous studies of Cry1J binding with FAW BBMVs [27,30]. Complete sharing of binding sites between Cry1Ja and Cry1A proteins was reported for *Spodoptera exigua* [23]. The sharing of Cry1Ab and Cry1Fa binding sites has been reported for several other lepidopteran species [22,31]. In addition, Cry1Ab and Cry1Fa have been shown to be cross-resistant against FAW [32].

The extent to which the shared Cry1Ja binding sites are involved in Cry1Ja toxicity is a critical determinant of potential cross-resistance with other proteins. This cross-resistance potential was clarified with the Cry1Ja bioassay data with wild-type and Cry1Fa-resistant FAW. These data showed that Cry1Ja was efficacious against both Cry1Fa-resistant and -susceptible FAW (Figure 4A,B). Therefore, binding at the Cry1Ja binding site is sufficient for achieving Cry1Ja toxicity and the loss of the Cry1A.88 and Cry1Fa binding site does not affect Cry1J toxicity. Finally, these data would indicate that full cross-resistance between Cry1J and Cry1Fa or Cry1Ab is unlikely.

The data presented here show that Cry1Ja has potential to be used as an insecticidal protein, but further work would be needed. One part of this work would involve the evaluation of shared binding sites between Cry1Ja and Cry1F in SBL and between Cry1Ja and Cry1Ab and Cry1Fa in CEW, with a required result that the Cry1Ja toxicity is not altered in resistant insects, as with Cry1Fa-resistant FAW. Another aspect to evaluate would be any possible effects Cry1Ja may have on the agronomic properties of Cry1Ja-expressing crop plants with a required outcome of none or limited agronomic effects. These results would show a strong case for the use of Cry1Ja as an insect control agent.

## 4. Conclusions

We have summarized the results of our competitive binding data in the form of a binding site model for Cry1Ja in these three insects (Figure 6). In SBL BBMVs, Cry1JP578V only shares binding sites with Cry1Fa but with Cry2A.127 and Vip3A. In CEW BBMVs, Cry1JP578V, Cry1Fa and Cry1A.88 share binding sites, while Cry2A.127 and Vip3A have completely independent binding sites. In FAW BBMVs, Cry1JP578V shares binding sites with Cry1A.88 and Cry1Fa, but also has an independent binding site that is not shared with Cry2A.127 and Vip3A. The model illustrates the diversity of the binding site relationships among different insects and reveals the potential for differing degrees of cross-resistance between proteins depending on the existence of unshared binding sites. Based on the model, there is some possible level of cross-resistance between Cry1Ja and Cry1F in SBL and between Cry1Ja and Cry1F or Cry1A in CEW and FAW. However, in FAW, Cry1J was shown to be efficacious against both Cry1Fa-resistant and -susceptible FAW. In addition, we have shown the in planta efficacity of Cry1J against SBL and FAW feeding. Our results add to the information reported in other studies that have evaluated the shared binding sites between Cry1Ja and Cry1A or Cry1Fa in order to understand the cross-resistance potential among these proteins in different Lepidoptera that cause crop damage [23,33], with this representing the first study to evaluate the binding site relationships between Cry1Ja and Cry1A, Cry1Fa, Cry2A or Vip3A in SBL. The results of the present study demonstrate the potential of Cry1Ja for development into a new insect control trait.

## 5. Materials and Methods

### 5.1. Protein Identification, Production and Preparation

A Cry1J-like protein (Cry1JDP166) was identified from a screen of Bt isolates from a proprietary internal collection from a strain designated DP166 [14] (GenBank Accession Number #OQ943180). Cry1JDP166 has 99.14% shared identity with Cry1Ja at the amino acid level. Cry1JDP166 was improved against proteolytic degradation and led to the development of Cry1JP578V [14]. Cry1JP578V was used in the competition binding assays due to its increased stability against proteolytic degradation.

Cry1JP578V without a crystal-forming domain (~71 kD) was expressed in an *E. coli* BL21(DE3) Gold cell expression system as follows. Cell pellets were resuspended in Buffer A (20 mM Tris pH 7.5, 500 mM NaCl and 5 mM imidazole) plus one tablet of cOmplete™ Protease Inhibitor Cocktail per 50 mL and 2.5 U/mL Pierce Universal Nuclease (Thermo Fisher Scientific Pierce Biotechnology, Rockford, IL, USA). The resuspended cell pellets were homogenized cells with two passes at 25 kpsi, followed by a 50 mL buffer A rinse. The supernatant was isolated by being centrifuged at 30,000× *g* for 20 min and filtered through a 0.45 µm filter. The filtered supernatant was passed over an Ni-NTA resin column washed with an increasing concentration of imidazole (0 to 20 mM) in buffer A and eluted with 125 mM imidazole in buffer A. The protein was further purified by anion exchange on a Hi Prep Q FF 16/10 anion exchange column (GE, Pittsburgh, PA, USA). The peak fractions were pooled, aliquoted and flash-frozen, and stored at −80 °C until needed.

Cry1JP578V was processed by trypsin (Millipore Sigma, St. Louis, MO, USA) digestion using a 1:10 ratio of trypsin to Cry1JP578V (1 μg trypsin for 10 μg Cry1JP578V) at 37 °C for one hour. Trypsin processing simulated the activity of the insect midgut proteases following ingestion of protoxin. Trypsin treatment yielded a stable protein fragment of approximately 62 kD when evaluated by SDS-PAGE. Purification of the trypsinized Cry1JP578V was achieved by anion exchange using a HiTrap Q FF column (GE, Pittsburgh, PA, USA). A small portion of purified, activated Cry1JP578V was labeled with Alexa fluor^®^ 488 (Thermo Fisher Scientific, Waltham, MA, USA) per the manufacturer’s protocol. The purified, activated and/or labeled Cry1JP578V proteins were quantified by SDS-PAGE gels stained with Simply Blue gel stain (ThermoFisher) that were imaged using a LAS4000 imager (GE Healthcare) and quantified by gel densitometry software (Phoretix 1D version 10.5, TotalLab, Ltd., Newcastle upon Tyne, UK) using BSA standards.

The Cry2A.127 and Cry1A.88 proteins were produced as previously stated in Liu et. al., 2019 [18]. The Vip3Ab protein was produced as previously stated in Zack et al., 2017 [34]. Cry1Fa and Cry1Ac were expressed in *Bacillus thuringiensis* strain BtG8 as follows. Cell pellets were lysed by resuspending in Buffer B (50 mM Tris pH 7.5, 300 mM NaCl, 2 mg/mL lysozyme, and 2.5 U/mL Benzonase Nuclease^®^) for 20 h @ 30 °C and 70 RPM lysate was centrifuged at 25,000× *g* for 30 min (F14-6x250y Rotor, 13,000 rpm). The pellets were washed five times with 1 M NaCl. The pellets were, then, washed two times with water. The pellets were stored at −20 °C. Each pellet was solubilized in 20 mL 50 mM Na_2_CO_3_, pH 11, 10 mM DTT for 15 min at 4 °C with stirring. The solubilized pellet was centrifuged at 26,000× *g* for 30 min. The supernatant containing solubilized proteins was further purified by ion exchange chromatography using a 5 mL HiTrap Q HP column equilibrated with 50 mM Na_2_CO_3_ (pH 11) and eluted with 50 mM Na_2_CO_3_ (pH 11) and 1 M NaCl. The fractions containing Cry1Fa were pooled and dialyzed again with 50 mM Na_2_CO_3_ (pH 11). The purified proteins were quantified by gel densitometry using BSA standards as described above.

The Cry1Ac, Cry1Fa and Cry2A.127 proteins were treated with 10:1, 5:1 and 1.3 trypsin ratio, respectively, to simulate insect midgut processing, leaving stable core fragments that were purified by anion exchange column chromatography (HiTrap Q FF 1 mL; GE Healthcare, Chicago, IL, USA) for Cry1Ac and Cry1Fa or by size filtration column chromatography (2 SuperDex 75 in tandem; GE Healthcare, Chicago, IL, USA) for Cry2A.127. Selected fractions were then combined and buffer exchanged into binding buffer by use of a Zeba desalting column (ThermoFisher, Waltham, MA, USA). The Vip3Ab and Cry1A.88 proteins were processed, and all the proteins were labelled with Alexafluor 488^®^ (Molecular Probes/ThermoFisher) following the manufacturer’s recommendations to prepare for the competition binding assays as previously described in Liu et al., 2019 [18]. For simplicity, the proteins labelled with Alexafluor 488^®^ are referred to using the prefix Alexa hyphenated to their name (e.g., Alexa-Cry1Ac).

### 5.2. BBMV Preparation

Brush border membrane vesicles (BBMVs) were isolated from gut tissue dissected from the penultimate instar of CEW, FAW and SBL larvae. To collect the gut tissue, larvae were pinned down through the prothoracic shield and the anal plate. A longitudinal incision was made through the cuticle along the dorsal side of the larvae to the last abdominal body segment. The food bolus was removed through an incision along the length of the midgut. The tissue was excised from the carcass by resecting at each foregut/midgut and midgut/hindgut junction. Fat bodies, Malpighian tubules, tracheae, and other non-midgut tissues were extracted from the midgut tissue during excision. The excised tissue was flash-frozen immediately in liquid nitrogen and then placed at −80 °C for long-term storage.

Frozen midgut tissue was used to produce brushed border membrane vesicles (BBMVs) by the differential magnesium precipitation method [35]. Briefly, midgut tissue was transferred to 30 mL Oakridge-style centrifuge tubes and homogenized in 9 volumes (weight/volume) ice-cold MET buffer (300 mM Mannitol (Millipore Sigma, St. Louis, MO, USA), 5 mM ethylene glycol-bis (2-aminoethylether)-N,N,N′,N′-tetra-acetic acid (EGTA) (Millipore Sigma, St. Louis, MO, USA), 17 mM Tris–HCl (pH 7.5) (Millipore Sigma, St. Louis, MO, USA) and cOmplete™ Protease Inhibitor Cocktail without EDTA for FAW and SBL and with EDTA for CEW (Roche, Mannheim, Germany)) with an Omni-TH homogenizer (Omni International, Kennesaw, GA, USA) on setting 5 for two 1 min periods, with one minute on ice between homogenizations. The homogenate was diluted with an equal volume of ice-cold 24 mM MgCl_2_, mixed and incubated on ice for 15 min. This mixture was centrifuged at 2500× *g* for 15 min at 4 °C to remove any cellular debris. The supernatant from the initial centrifugation was further centrifuged at 30,000× *g* for 30 min at 4 °C. The resulting pellet was resuspended in 0.5× homogenate volume of ice-cold MET buffer. An equal volume of ice-cold 24 mM MgCl_2_ (Millipore Sigma, St. Louis, MO, USA) was added, the solution was mixed and incubated on ice for 15 min. Another low-speed centrifugation 2500× *g* for 15 min at 4 °C was performed. The resulting supernatant was further centrifugated at 30,000× *g* for 30 min at 4 °C. The resulting BBMV pellet was resuspended in buffer (10 mM HEPES (Millipore Sigma, St. Louis, MO, USA), pH 7.4, 150 mM NaCl (Millipore Sigma, St. Louis, MO, USA)), flash-frozen in liquid nitrogen, and stored at −80 °C.

The protein concentration of the BBMV preparations was determined by the bicinchoninic acid method [36]. Alkaline phosphatase and amino peptidase activities were used as marker enzymes to track the purification of the brush border membrane from preparation to preparation. Alkaline phosphatase assays were performed in a diethanolamine buffer (10% *v*/*v* diethanolamine (Millipore Sigma, St. Louis, MO, USA), 0.5 mM MgCl_2_ pH 9.8)) with Alkaline Phosphatase Substrate (Bio-Rad, Hercules, CA, USA). Amino peptidase assays were performed in a TRIS buffer (25 mM NaCl, 20 mM Tris-HCl pH 8) with L-leucine-p-nitroanilide substrate (Millipore Sigma, St. Louis, MO, USA). Enzymatic activity was measured in a Spectramax M2 (Molecular Devices, San Jose, CA, USA) at 405 nm every 30 s during the initial 5 min of the reactions.

### 5.3. Competition Binding Assays

Binding assays were performed using one of two buffers: 50 mM Na_2_CO_3_/HCl, 150 mM NaCl and 0.1% Tween-20, pH 9.6 with or without 2× cOmplete™ Protease Inhibitor Cocktail without EDTA (Roche, Mannheim, Germany) or 20mM Na_2_CO_3_/HCl pH 9.6, 100 mM NaCl, 0.2% Tween-20 with or without 2× cOmplete™ Protease Inhibitor Cocktail without EDTA (Roche, Mannheim, Germany), as determined empirically. The conditions were further optimized for each protein and insect combination by varying the amount of BBMVs and concentration of Alexa-labeled protein as follows: 5 nM Alexa Cry1JP578V and 30 μg CEW BBMVs; 5 nM Alexa Cry1JP578V and 10 μg FAW BBMVs; 2.5 nM Alexa Cry1JP578V and 30 μg SBL BBMVs; 2.5 nM Alexa Cry1A.88 and 25 μg CEW BBMVs; 2.5 nM Alexa Cry1A.88 and 25 μg FAW BBMVs; 50 nM Alexa Cry1Ac and 5 μg SBL BBMVs; 100 nM Alexa Cry1Fa and 1 μg CEW BBMVs; 10 nM Alexa Cry1Fa and 40 μg FAW BBMVs; 5 nM Alexa Cry1Fa and 60 μg SBL BBMVs; 10 nM Alexa Cry2A.127 and 60 μg CEW BBMVs; 10 nM Alexa Cry2A.127 and 40 μg FAW BBMVs; 20 nM Alexa Cry2A.127 and 30 μg SBL BBMVs; 5 nM Alexa Vip3Ab and 20 μg CEW BBMVs; 10 nM Alexa Vip3Ab and 20 μg FAW BBMVs; 5 nM Alexa Vip3Ab and 40 μg SBL BBMVs. To demonstrate specific binding, the BBMVs were incubated with an Alexa-labeled protein in binding buffer (total volume of 100 µL) for one hour with shaking at room temperature in the absence or presence of different concentrations of unlabeled Cry1JP578V or another protein competitor.

After incubation, the samples were centrifuged at 20,817× *g* for 10 min at 4 °C to separate the BBMVs with bound proteins from the unbound proteins. The BBMV/bound protein pellets were washed twice with 1 ml of ice-cold binding buffer with centrifugation (20,817× *g* for 10 min at 4 °C) to further eliminate any remaining unbound protein. The final pellet was solubilized in 1× LDS reducing sample buffer (Invitrogen, Carlsbad, CA, USA), heated to 100 °C for 10 min and subjected to SDS-PAGE using 4–12% Bis-Tris polyacrylamide gels (Life Technologies, Waltham, MA, USA). The amount of Alexa-labeled protein in the gel from each sample was captured by a digital fluorescence imaging system (Typhoon FLA 9500, GE Healthcare, Chicago, IL, USA). The digitized images were analyzed by densitometry software (Phoretix 1D, TotalLab, Ltd., Newcastle upon Tyne, UK). The data analysis was performed by setting “Total” binding as the binding in the absence of any competitors and expressing the extent of the competition as a percentage of the “Total” binding with a competitor present. The homologous competition curves were fitted using the Sigmoidal dose-response (variable slope) equation in Prism analysis and graphing software (GraphPad Software version 10.3.1, San Diego, CA, USA). The heterologous competition assays were statistically analyzed by one-way ANOVA with Tukey’s post hoc test for Alexa Cry1JP578V and with one-way ANOVA with Sidak’s post hoc test for the reciprocal heterologous competition of Cry1JP578V using Prism analysis and graphing software (GraphPad Software, San Diego, CA, USA).

### 5.4. In Planta Efficacy Evaluation

The Cry1JDP166 sequence was modified to introduce three amino acid changes: glutamate to aspartate at position 115, alanine to valine at position 594 and lysine to isoleucine at position 724, and the resulting polypeptide was named Cry1JPS1 [14]. Soybean plants expressing Cry1JPS1 under the control of an Arabidopsis promoter [37] were produced from immature seed cultures following the Agrobacterium-mediated transformation protocol [38,39,40,41]. Briefly, immature seeds were harvested from soybean pods of plants grown in the greenhouse under standard conditions. The seeds were surface sterilized, the immature cotyledons were aseptically excised and the cultures were maintained in 250 mL flasks containing 50 mL of liquid media on rotary shakers at 26 °C under cool white fluorescent lights with a 16/8 h day/night photoperiod [38,41]. Agrobacterium tumefaciens carrying plasmids with genes of interest were used to transform the immature cotyledons. Transgenic events were selected and regenerated to maturity. These plants were grown under the same conditions as the wild-type plants but in separate growth chambers.

Insect control evaluation in soybean plants was performed as described previously [18] using 17 T1 homozygous plants. The protein accumulation levels for Cry1JPS1 in leaf tissue were quantified through mass spectrometry detecting the peptide sequence ILDGLIEANIPSFR, as described previously [17]. The T1 bioassay was statistically analyzed by one sample test using Prism analysis and graphing software (GraphPad Software, San Diego, CA, USA).

### 5.5. Insect Bioassays

An FAW population highly resistant to Cry1Fa (>300-fold resistant ratio) collected from Puerto Rico in 2009 [16] was used to evaluate the cross-resistance between Cry1JP578V and Cry1Fa. A Cry1Fa-susceptible FAW population was used as a reference control population in the bioassays. The purified recombinant Cry1JP578V protein variant, Cry1J PS1, was evaluated at seven concentrations ranging from 0.7 to 700 µg/mL. The bioassays were conducted in 96-well plates by mixing 25 µL of the respective test concentration with 75 µL of the Southland Multiple Species Diet (Southland Products Incorporated, Lake Village, AK, USA). A plain diet treated only with carbonate buffer (pH 10.0) was kept as a control. A single larva (<16 h old first instar) was placed in each well. The bioassay was conducted with 32 replicates for each concentration (n = 32 larvae) and was repeated four times for each insect population. The bioassay plates were held in an incubator at 27 °C, 65 ± 5% RH. Larval mortality was observed at 4 days after treatment. Larvae were considered dead if they did not move when they were probed using a camel hairbrush. Live larvae were collected for measuring the body weight. The total numbers of dead larvae were used to calculate the lethal concentration affecting 50% of the larvae (LC_50_), and the weight of the live larvae were used to calculate the growth inhibition concentration affecting 50% of the larvae (EC_50_). Statistical analyses were conducted using SAS software, Version 9.4. To estimate the LC_50_, SAS PROC PROBIT was used to fit the probit model for each bioassay of each population. The probit model used was $\mathrm{Pr}\left(mortality\right)=C+\left(1-C\right)F\left(x^{\prime }\beta \right)$, where $x^{\prime }$ is a vector of the base-10 logarithm of the tested concentrations. For each bioassay, if the observed baseline mortality rate was zero, then the natural mortality, denoted as C above, was fixed at zero; otherwise, natural mortality was estimated and used in fitting the probit model. To estimate the EC_50_, the growth inhibition at each concentration was calculated as a percentage of the weight reduction compared to the weight at concentration 0 (control plain diet) for each bioassay of each population. SAS PROC NLMIXED was used to fit the Emax model, i.e., a three-parameter logistic model, for the growth inhibition data.

$$y_{i}=\mu_{i}+\varepsilon_{i}=E_{\max}-\frac{E_{\max}}{1+{\left(\frac{D_{i}}{EC_{50}}\right)}^{slope}}+\varepsilon_{i}$$

*y_i_* = the observed growth inhibition at the *i*th dosage level; *μ_i_* = the expected growth inhibition at the *i*th dosage level; *E_max_* = the maximum growth inhibition at the infinite dosage level (upper asymptote); *D_i_* = the dosage for the *i*th dosage level slope = the slope factor (Hill factor), measuring the sensitivity of the response to the concentration range, determining the steepness of the concentration-response curve (slope > 0 for an ascending curve); ε_i_ = the random error term associated with the *i*th dosage level. The error terms are normally distributed with a mean of zero and a variance of *σ*^2^. The model parameter estimates and their standard errors (SEs) were obtained using the maximum likelihood method. The parameter space of *E_max_* was set to be less than 100% and the parameter space of *σ*^2^ was set to be positive. A random-effects meta-analysis approach [42] was utilized to derive the estimate of the overall mean estimate of the LC_50_ and EC_50_ across the independent bioassays for each population. SAS PROC MIXED was utilized to obtain the estimates.

## Data Availability

The original contributions presented in the study are included in the article/Supplementary Material, further inquiries can be directed to the corresponding authors.

## Supplementary Materials

The following supporting information can be downloaded at: https://www.mdpi.com/article/10.3390/toxins16090384/s1, Figure S1: Homology models of Cry1J P578 and P578V.
