# Supplementary material for: Utility of Cry1Ja for Transgenic Insect Control
Source: Toxins (Basel). 2024 Sep 4;16(9):384. doi: 10.3390/toxins16090384 (PMC11435796; doi:10.3390/toxins16090384)
Supplement: Supplementary file 1 [file toxins-16-00384-s001.zip › toxins-3165519-supplementary.pdf]

Utility of Cry1Ja for Transgenic Insect Control

John P. Mathis, Catherine Clark, Amit Sethi, Benchie Ortegon, Gilda Rauscher, Russ Booth, Samuel Coder and Mark E. Nelson

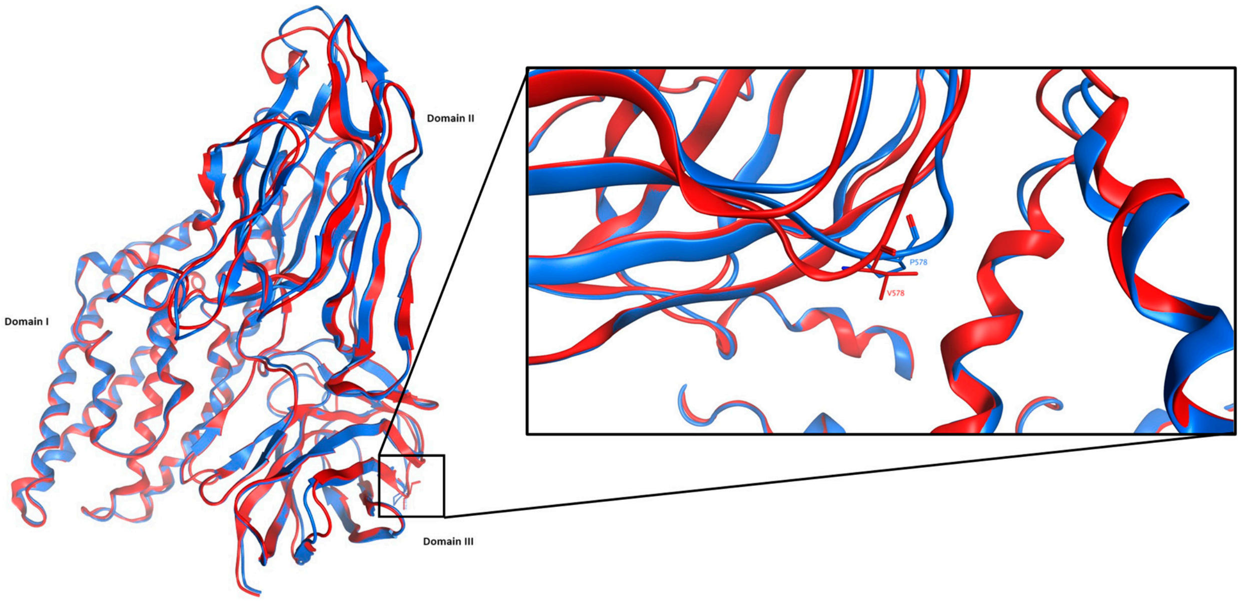

Figure S1. Homology models of Cry1J P578 and P578V. Cry1J P578 (blue) and P578V (red) models were made in the Molecular Operating Environment (MOE) software (version 2024.06 Chemical Computing Group ULC, 1010 Sherbrooke St. West, Suite #910, Montreal, QC, Canada, H3A 2R7, 2024) using the structure of Cry1A (PDB ID 8w7n) as a template. Ten models for each variant were calculated, minimized, and scored using the MOE standard parameters. The best scoring intermediate model based on the Generalized Born/Volume Integral (GB/VI) was chosen and displayed. (Labute 2008)

Disclaimer/Publisher's Note: The statements, opinions and data contained in all publications are solely those of the individual author(s) and contributor(s) and not of MDPI and/or the editor(s). MDPI and/or the editor(s) disclaim responsibility for any injury to people or property resulting from any ideas, methods, instructions or products referred to in the content.
